# Occipital nerve blocks: a promising approach for chronic migraines?

**DOI:** 10.1097/MS9.0000000000001443

**Published:** 2023-11-01

**Authors:** Kanza Farhan, Muhammad Ashbar Wadood, Jaiwanti Kumari, Muhammad Burhan Tariq, Aliza Ahmed, Abdul-Samed Mohammed, Mubarick Nungbaso Asumah

**Affiliations:** a Sindh Medical College, Jinnah Sindh Medical University, Karachi, Pakistan; b School of Public Health, University for Development Studies; c Ministry of Health, Nurses’ and Midwives’ Training College, Tamale, Northern Region, Ghana

*Dear Editor,*

The most prevalent debilitating brain condition is migraine. Chronic migraine, which manifests as having a migrainous headache at least 15 days a month, is quite incapacitating^1,2^. The 30th biggest cause of disability-adjusted life years and nearly 1% of all disability-adjusted life years worldwide, according to the Global Burden of Disease 2010 report, is migraine. Migraines have significant personal and societal costs^3^. The growing interest in the pathogenesis, epidemiology, and therapy of migraine has been sparked by the idea that it can be a chronic disorder^1^. A logical course of action is to seek specialized care when frequent and severe headaches make it difficult to function on a daily basis. Referring patients to headache clinics enables thorough evaluation and takes greater occipital nerve blocks (GON-blocks) into account as potential treatments if the primary line of treatment is not working^4^. This letter highlights the possible advantages of GON-blocks as a promising alternative therapy, eventually seeking to enhance the quality of life for patients who experience chronic migraines.

Global estimates of the prevalence of chronic migraine range from 0 to 5.1%, with the majority of studies including general populations finding a prevalence of 1.4–2.2%^5^. According to a major population-based study, 57% of persons with chronic migraine missed at least 5 days of work or school over a 3-month period^3^. Patients who suffer from chronic migraines bear a heavy burden because of their frequent headaches, heightened sensitivity to visual, aural, and olfactory stimuli, nausea, and vomiting. Through both direct and indirect medical costs, it also has an impact on society^3^.

GON-block has become a popular alternative treatment for many primary and secondary headache conditions^4^. The rationale for using GON-block for acute and preventive treatment in various headache disorders is that GON-block targets the anatomical and functional continuum between trigeminal and cervical fibers in the so-called trigeminocervical complex^6^. With a cutaneous distribution across the back of the head, the greater occipital nerve (GON) transmits sensory fibers that primarily begin at C2. Therefore, GON-blocks may reduce neuronal hyperexcitability at the second-order neuron level, hence lowering pain in the trigeminal area^7^. GON-block also helped with anxiety, depression, and the sleep quality index. The connection between the ophthalmic branch of the trigeminal nerve and the modification of the afferent pathway to the trigeminal nucleus caudalis and the greater occipital nerve is assumed to be the basis for the proposed mechanism for the effect of the GON block on lowering migraine symptoms^6^.

GON-block treatment safety and efficacy have been evaluated and determined to be satisfactory, with occasional and transient emergent side effects^4^. These adverse side effects included displeasure at the site of injection, a rise in the severity of headaches, and an unpleasant sensation at the back of the head in a subset of individuals^8^. Patients selected to undergo occipital nerve blocks should fit the diagnosis criteria for migraine headaches as outlined by the International Classification of Headache Disorders (ICHD-3 Beta) recommendations. Patients with documented allergies to lidocaine, pregnant individuals, those with a history of psychosis, depression, or dementia, as well as patients who have used migraine-preventative medication within the past 3 months, are usually excluded^7^.

In conclusion, chronic migraines place a significant strain on both individuals and society. GON-blocks show potential as a treatment strategy because they can target the trigeminocervical complex and lessen neuronal hyperexcitability and GON-blocks. GON-blocks are a promising option for enhancing the quality of life for chronic migraine sufferers despite the occasional adverse effects that may occur. Additional studies and clinical applications could considerably help people dealing with this crippling ailment.

## Ethical approval

No ethical approval was required for this editorial manuscript.

## Consent

An informed consent was not required for this manuscript because it is an editorial piece.

## Sources of funding

No funds, no grants, or other support was received.

## Author contribution

K.F. and A.A.: conceptualization; K.F.: funding acquisition; M.N.A.: investigation; B.T. and A.A.: project administration; K.F., B.T., and A.A.: resources; M.N.A.: software; K.F.: supervision; K.F.: visualization; J.K. and M.A.W.: writing – original draft; A.A., B.T., and M.N.A.: writing – review and editing. All authors were involved in the validation and final approval of the manuscript for publication.

## Conflicts of interest disclosure

There are no conflicts of interest.

## Research registration unique identifying number (UIN)

Not applicable.

## Guarantor

All authors.

## Data availability statement

No data were generated for this manuscript.

## Provenance and peer review

Not commissioned, externally peer-reviewed.
